# Speech- and Text-Based Emotion Recognition in Anesthesiology Residents During Critical Incident Simulation Training: Exploratory Observational Study

**DOI:** 10.2196/96628

**Published:** 2026-08-21

**Authors:** Sapir Gershov, Itay Bentov, Fadi Mahameed, Aeyal Raz, Shlomi Laufer

**Affiliations:** 1 Department of Psychiatry, NYU Grossman School of Medicine, 1 Park Ave., 8th Floor, New York, NY, 10016, United States, 1 646-934-4738; 2 Technion Autonomous Systems Program, Technion – Israel Institute of Technology, Haifa, Israel; 3 Department of Anesthesiology and Pain Medicine, Harborview Medical Center, Seattle, WA, United States; 4 Department of Anesthesiology, Rambam Health Care Campus, Haifa, Israel; 5 Faculty of Data and Decision Sciences, Technion – Israel Institute of Technology, Haifa, Israel; 6 Rappaport Faculty of Medicine, Technion – Israel Institute of Technology, Haifa, Israel

**Keywords:** simulation-based medical education, critical incident simulation, affective communication, speech-based emotion recognition, text-based emotion recognition, anesthesiology residents, formative feedback

## Abstract

**Background:**

Communication during crisis management involves not only the exchange of clinical information but also the expression and regulation of emotional tone and sentiment, which may reflect clinician performance during a critical incident. In simulation-based medical education, these emotional dynamics are rarely measured objectively, limiting the ability to capture aspects of performance relevant to competency assessment.

**Objective:**

This study aimed to examine whether automatically extracted speech- and text-based emotion signals varied across predefined phases of critical incident simulation training and whether these signals were associated with expert-rated performance among anesthesiology residents.

**Methods:**

In this exploratory observational study, we analyzed 164 simulated crisis scenarios performed by 90 anesthesiology residents from 17 hospitals during a national board preparation workshop. Each high-fidelity simulation comprised 4 predefined phases: initial condition, extreme deterioration, advanced cardiovascular life support (ACLS) management, and recovery. Resident speech was isolated and analyzed using a speech-based emotion recognition (SER) model that estimated 8 emotion categories per utterance. Speech was also automatically transcribed and analyzed using text-based emotion recognition (TER), which estimated 9 emotion categories. Expert anesthesiologists rated performance on a 1-5 scale. ANOVA tested associations between emotion categories and performance, as well as variation across simulation phases. Exploratory mixed-effects sensitivity analyses accounted for repeated simulations and scenario-level clustering.

**Results:**

After 7 recordings were excluded because of technical issues, 65 of 164 (39.6%) simulations were classified as poor performance (score ≤2), 72 (43.9%) as intermediate, and 27 (16.5%) as excellent (score=5). SER categories differed across performance groups (*F*_2,161_=4.61; *P*=.02): poor performance showed more fear and surprise, whereas excellent performance showed more calm, happiness, and neutrality. In mixed-effects analyses, performance group remained associated with happy, angry, fearful, surprise, calm, and neutral speech-emotion proportions after false discovery rate correction. The largest excellent vs poor differences were observed for neutral (+4.24 percentage points), happy (+4.11), and surprise (−3.17) speech affect. TER showed a directionally similar but nonsignificant omnibus association (*F*_2,161_=2.57; *P*=.08); mixed-effects analyses identified associations with happy and sad text-emotion proportions after correction. During extreme deterioration and ACLS management, fear and surprise together accounted for 64.1% and 64.6% of classified speech emotions, respectively, among poor performances. During ACLS management, neutral and calm emotions accounted for 49.1% and 37.7%, respectively, among excellent performances.

**Conclusions:**

Automated speech- and text-based emotion recognition captured phase-dependent affective communication patterns during critical incident simulations. Speech-derived features were more consistently associated with expert-rated performance, whereas text-derived findings were more exploratory and emotion-specific. These markers may complement simulation debriefing and formative feedback, but validation using multirater outcomes, preregistered adjusted models, and larger multicenter datasets is needed before they inform competency assessment.

## Introduction

### Affective Communication in Critical Care

Effective communication is central to safe care in high acuity clinical environments, such as in the operating room (OR) and intensive care unit (ICU). During critical events, clinicians must rapidly interpret evolving information, issue clear instructions, coordinate team responses, and maintain situational awareness under pressure. These exchanges are not purely informational. They also convey affective signals, including urgency, confidence, calmness, hesitation, and distress, which may influence team coordination, decision-making, and clinical execution [1,2]. Prior work has shown that both communication quality [3] and emotional processes [4] are relevant to performance in acute care settings.

In anesthesiology, these issues are especially complex because anesthesiologists operate under sustained cognitive load. Stress and arousal are expected components of medical emergencies, but when poorly regulated, they may impair attention, communication, and decision-making [5-7]. This is particularly challenging in perioperative crises, which often elicit strong emotional responses and a need for structured debriefing or recovery time [6].

Emotion and communication are intertwined rather than interchangeable. Well-regulated affect can facilitate clarity, closed-loop confirmation, and team coordination; dysregulated affect may coincide with interruptions, hesitations, or missed confirmations [7-9]. The key question is therefore not whether emotion is present, but how affective patterns unfold during critical situations and whether they align with effective communication behaviors and clinical performance.

### Simulation-Based Assessment and the Need for Objective Communication Markers

Simulation-based crisis incident management training has been extensively used by anesthesiologists for over half a century [10,11], as it is effective for skill acquisition [12] and knowledge retention [13]. Critical incident simulation training is commonly used in high acuity training [14-16] and can also provide an opportunity to improve patient safety by exploring providers’ responses to stressful situations [17,18]. Simulation-based assessment (SBA) is now a widely accepted method for evaluating clinical competency and is incorporated into board certification examinations by professional societies, including the Israel Society of Anesthesiologists [18]. However, current SBA approaches emphasize observable behavior scored by expert evaluators, which can be vulnerable to bias [19,20], while the emotional dimensions of communication remain largely unexamined. This creates a need for complementary, objective measures that can enrich traditional assessments without replacing expert evaluation.

### Speech- and Text-Based Emotion Recognition

Recent advances in natural language processing (NLP) and speech analysis have created new opportunities to quantify emotional content in spoken clinical communication. Text-based emotion recognition (TER) [21,22] can infer emotional meaning from transcribed language, whereas speech-based emotion recognition (SER) [23,24] captures paralinguistic features such as tone, intensity, and vocal affect. The integration of these techniques provides a multidimensional view of communication, extending analysis beyond spoken words to encompass tone, urgency, and affect [25,26]. At the same time, applying TER and SER in healthcare poses challenges due to the specialized vocabulary and context-dependent nature of clinical communication [27]. Recent work has also emphasized the broader movement toward technology-enhanced assessment of nontechnical skills in high acuity procedural environments. For example, digital approaches to assessing nontechnical skills in the operating room have been proposed to provide more objective, scalable, and timely feedback, although validation and interpretability remain major challenges [7]. Similarly, recent reviews of SER [25] and textual emotion detection [28] in healthcare highlight the promise of automated affective analysis while emphasizing the need for careful domain adaptation, transparent evaluation, and validation in clinically meaningful settings. These considerations are particularly relevant in simulation-based anesthesiology training, where communication is brief, protocol-driven, emotionally charged, and shaped by clinical context. Accordingly, SER and TER should be viewed as potential complementary markers of affective communication rather than direct measures of clinical competency.

The contribution of the present study is the integration of existing speech- and text-based emotion recognition methods within a phase-structured, high-fidelity anesthesiology simulation setting. Specifically, we aligned resident-specific affective communication signals with predefined crisis phases and expert-rated simulation performance. This design allowed us to examine whether automatically derived emotion markers provide complementary information about affective communication during simulation-based training.

### Study Aim and Hypotheses

In this study, we conceptualized affective communication as a behavioral signal that may reflect several overlapping processes relevant to crisis management, including cognitive load, stress regulation, communication clarity, situational awareness, and individual communication style. These processes are related to, but not equivalent to, clinical competency. Consistent with this conceptual framing, emotion recognition outputs were treated as exploratory markers of affective communication rather than direct measures of clinical competency. Specifically, our aim was to automatically extract emotion signals from speech and transcribe text associated with expert-rated performance during simulation-based critical incident training among anesthesiology residents. Using audio recordings from simulated ICU scenarios, we analyzed SER and text-based emotion recognition outputs across predefined phases of each simulation. We hypothesized that higher-performing residents would display a greater level of regulated affective patterns, reflected in calmer or more positive emotional signals, whereas lower-performing residents would display a greater level of negative and high arousal emotional patterns. We also examined whether these emotional patterns differed across simulation phases, particularly during the most demanding portions of the scenario.

## Methods

### Study Design

This exploratory observational study examined whether automatically extracted emotion signals from residents’ speech and transcribed language were associated with expert-rated performance during simulation-based critical incident training in anesthesiology.

### Setting and Participants

Anesthesiology residency in Israel, as in other countries [29], includes staged qualifying examinations throughout training. In the first stage, residents must pass a national written board exam before advancing to the senior phase of residency. To become an attending anesthesiologist, residents must demonstrate knowledge and competency through oral board examinations and simulated scenarios. The Israel Society of Anesthesiologists organizes a biannual, 2-day workshop to prepare senior anesthesiology residents for their final board examinations. This workshop includes hands-on practice with high-fidelity simulations followed by expert feedback from board-certified anesthesiologists.

For this study, we analyzed all the residents who participated in the workshops conducted in 2022 and 2023. During enrollment, residents completed a demographic questionnaire that included their level of clinical experience, measured in years of residency, and the number of prior SBAs they had completed.

### Ethical Considerations

The Rambam Health Care Campus Institutional Review Board approved this study (0482‐20-RMB). All participants provided written informed consent before participation.

### Simulation Procedures and Performance Ratings

An experienced anesthesiologist and a medical simulation specialist developed 7 clinical simulation scenarios in accordance with advanced cardiovascular life support (ACLS) and advanced trauma life support (ATLS) principles: (1) severe anaphylaxis reaction, (2) postoperative severe bradycardia, (3) postoperative opioid overdose, (4) postoperative hypoglycemia, (5) postoperative residual paralysis, (6) postoperative supraventricular tachycardia, and (7) patient with head trauma.

Each resident was randomly assigned to 2 simulations. During each simulation, 2 members of the research team played the roles of a nurse and a medical intern, respectively. A board-certified anesthesiologist evaluated the resident’s clinical performance using a task-specific checklist. The simulation setup included a Laerdal full-body manikin to create a high-fidelity, realistic environment (see Figure 1). The simulation area was recorded from multiple angles, and to ensure clear audio recordings, all participants (residents and team members) were equipped with wireless lavalier microphones. The data was recorded and synchronized using StreamPix digital video recording software from NorPix Inc.

To better capture participants’ expressed emotions, we divided the simulation timeline into 4 chronologically distinct phases:

1. Initial condition: Participants understand the patient’s status by examining the patient and communicating with the nurse.
2. Extreme deterioration: The patient’s condition has deteriorated significantly (eg, dropping blood pressure, rising heart rate). Participants must manage this crisis and respond appropriately.
3. ACLS management: Participants must execute procedures quickly and accurately. Additionally, participants must demonstrate confidence and familiarity with the protocols. In the case of simulation 7 (patient with a head trauma), residents performed and managed ATLS.
4. Recovery: Participants update the team on the patient’s status and coordinate postcrisis care.

This phase-based structure enabled us to examine whether emotional expression varied across different levels of clinical demand within the scenario. Phases were controlled by the simulator operator, each bounded by start and end timestamps and observable clinical cues. These included changes displayed on the patient monitor, such as alterations in vital signs and alarm activations, and, in some scenarios, physical indicators on the manikin itself (eg, breathing sounds, airway obstruction, chest movement, pupil size, etc).

At the end of each simulation, the anesthesiologist evaluator provided an overall performance rating on a Likert scale of 1 to 5, with 1 indicating poor performance and 5 indicating excellent performance. Based on these scores, we divided our sample into the following groups: poor performance (evaluation scores ≤2), intermediate performance (evaluation scores 3‐4), and excellent performance (evaluation scores=5).

**Figure 1.**
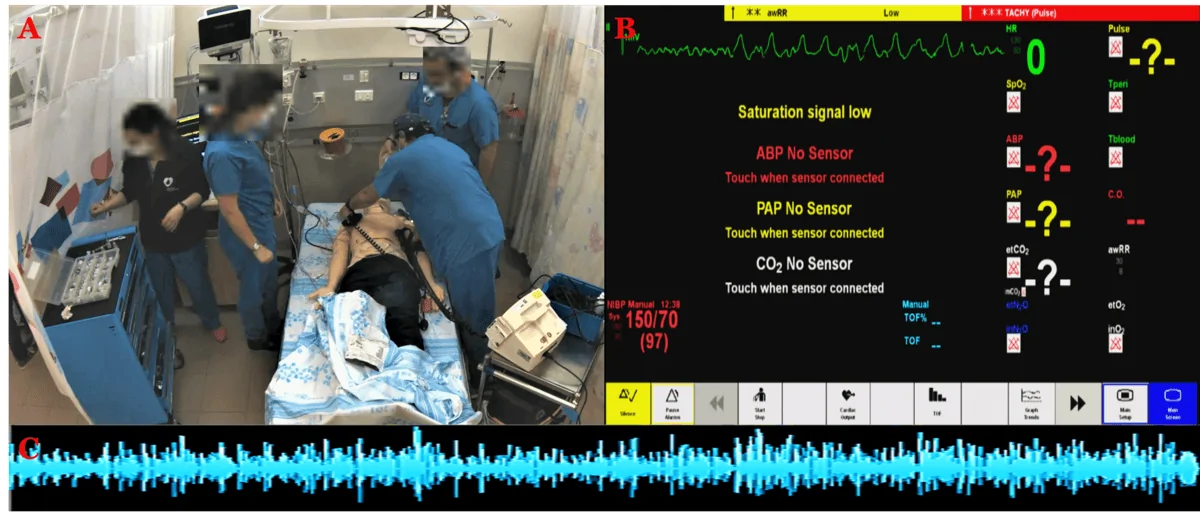
Simulation environment setup. (A) General overview of the simulation area. The faces have been manually blurred; (B) patient monitor display; (C) illustration of the lavalier microphone audio levels.

### Audio Collection and Preprocessing

To minimize the effect of mixed audio recordings, participants (resident, nurse, and intern) were recorded on separate audio channels. However, participants’ speech overlap and background noise (eg, patient monitor alarms) disrupted speech coherence, prompting us to integrate a speech-cleaning framework called end-to-end neural diarization with speaker subspace (EEND-SS) [30]. This framework performs several speech processing procedures (speaker diarization, speech separation, and speaker counting) and produces 2 types of audio files from each raw audio channel, (1) single-speaker files for each participant and (2) background noise files devoid of any traces of speech. These procedures eliminate disturbances that could have affected speech analysis. Additionally, the EEND-SS framework generates segments of “silence” areas in all processed audio files; silence thresholds are fine-tuned to optimize the results (see Figure 2A).

**Figure 2.**
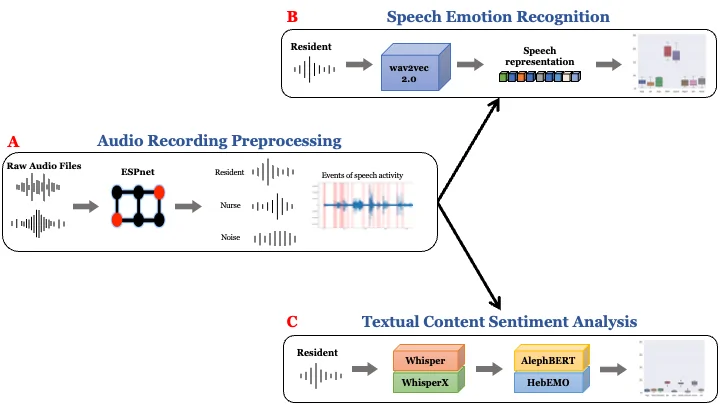
Illustration of the text-based emotion recognition (TER) and speech-based emotion recognition (SER) pipeline. (A) Preprocessing of the raw audio channels via ESPnet framework to extract intervals of the resident verbal expressions; (B) based on the extracted resident speech intervals, we executed SER for every sentence; (C) based on the extracted resident speech intervals, we transcribed each sentence and executed TER.

### Speech-Based Emotion Recognition

Each EEND-SS single-speaker file is labeled according to the audio source (ie, anesthesia resident, nurse, and medical intern). The following stages apply only to the anesthesia resident’s single-speaker audio file. First, feature extraction was performed using the SpeechBrain toolkit [31]. Specifically, we applied the wav2vec 2.0 model [32], which was pretrained via self supervision. Then, we utilized Pepino et al’s [33] model, which generates emotion category probabilities for 8 classes: happy, sad, angry, fearful, surprised, disgusted, calm, and neutral (see Figure 2B). During inference, the predicted emotion category for each segment was defined as the class with the highest probability. Segment-level predictions were then aggregated within each simulation, performance group, and simulation phase to estimate the relative frequency of each emotion category. These aggregated proportions were used in the statistical analyses.

### Text-Based Emotion Recognition

The processed anesthesia resident audio file was transcribed to Hebrew using a state-of-the-art, large-scale, weakly supervised speech recognition model, Whisper [34]. In addition, since Whisper only provides timestamps for each speech utterance, we used the work of Bain et al [35] to generate a word-level timestamped transcription. Afterward, we performed word- and sentence-level TER using 2 language models trained specifically on Hebrew text: AlephBERT [36] and HebEMO [37] (see Figure 2C). As with the speech-based model, the dominant text-based emotion category was defined as the class with the highest model score for a given utterance. To improve compatibility with clinical terminology, we applied the medical domain adaptation procedure described in our previous work [38], which involved adapting the text-processing pipeline to medical language. We did not fine-tune the emotion recognition models on the current performance labels, thereby avoiding leakage between performance ratings and emotion classification outputs.

### Implementation Details

All analyses were conducted on a computing cluster with 2 NVIDIA RTX A6000 48 GB graphics processing units (GPUs). The software environment consisted of Ubuntu 20.04 LTS, Python (version 3.9), and PyTorch (version 2.00). The EEND-SS framework used 6 transformer encoders and Conv-TasNet [39], which incorporates 8 temporal convolutional network blocks. The wav2vec 2.0 model had a feature encoder with 7 convolutional neural network layers and a transformer with 12 encoder layers. Both AlephBERT and HebEMO were used with their default parameters.

### Statistical Analysis

To evaluate whether the distribution of emotions differed across performance groups, we first used one-way ANOVA as an exploratory omnibus test. All statistical tests were 2-sided. Statistical significance was defined as *P*<.05, and exact *P* values are reported unless *P*<.001. Because residents could contribute more than 1 simulation and simulations differed by scenario, we then conducted exploratory mixed-effects sensitivity analyses. Emotion proportions were modeled separately for each emotion category. The performance group was included as the primary fixed effect, with random intercepts for resident and simulation scenario. Residency year and sex were included as covariates when available. Global performance-group effects were evaluated using likelihood ratio tests comparing models with and without performance group. False discovery rate (FDR) correction was applied across emotion-level tests within each modality. These sensitivity analyses were intended to assess whether the observed associations persisted after accounting for repeated observations and scenario-level clustering.

We also performed phase-stratified analyses to examine whether the relationship between emotional expression and performance varied across phases of the simulation scenario. All statistical analyses were executed using SciPy (version 1.16.1) and statsmodels (version 0.14.6).

## Results

### Sample Characteristics

We recruited 90 senior anesthesiology residents from 17 different hospitals, representing a diverse range of clinical backgrounds (see Table 1). On average, each resident participated in 2 different SBAs, yielding 171 recordings overall. Seven simulation recordings were excluded due to technical issues, including incomplete audio or video capture and severe audio artifacts, resulting in the final analytic sample of 164 simulations (see Table 2).

Based on expert assessors’ ratings, 65 out of 164 simulations (39.6%) were classified as poor performance (scores ≤2), 72 out of 164 (43.9%) as intermediate performance (scores 3‐4), and 27 out of 164 (16.5%) as excellent performance (score=5). The final dataset included recordings from all 7 simulation scenarios, with the largest proportions contributed by postoperative residual paralysis (42 out of 164 simulations, 25.6%) and postoperative severe hypoglycemia (36 out of 164 simulations, 22.0%) scenarios. It is worth noting that although participants communicated in Hebrew (with some English terms), the EEND-SS framework successfully processed our participants’ speech.

**Table 1.** Demographic questionnaire summary.

Characteristic	Residents (N=90), n (%)	Years of residency, mean (SD)
Sex		
Male	58 (64)	5.48 (1.32)
Female	32 (36)	5.06 (3.51)
Residency year		
4	26 (29)	—^a^
5	32 (36)	—
6	19 (21)	—
≥7	13 (14)	—

a Not applicable.

**Table 2.** Simulation dataset summary.

Simulation type	Simulations (N=164), n (%)	Performance score, n				
		1	2	3	4	5
Patient with a severe anaphylaxis reaction	24 (14.6)	2	5	7	6	4
Postoperative patient with severe bradycardia	26 (15.9)	5	4	4	10	3
Postoperative patient with opioid overdose	12 (7.3)	1	2	2	4	3
Postoperative patient with severe hypoglycemia	36 (22.0)	7	7	6	8	8
Postoperative patient with residual paralysis	42 (25.6)	7	12	9	7	7
Postoperative patient with severe supraventricular tachycardia	14 (8.5)	5	2	4	2	1
Patient with head trauma	10 (6.1)	4	2	2	1	1

### Affective Communication Across Performance Groups

Across performance groups, SER emotion proportions differed significantly (*F*_2,161_=4.61; *P*=.02; Figure 3). Poor performances (scores ≤2) showed higher levels of fear and surprise, whereas excellent performances (score=5) showed higher levels of calm and happiness. TER emotion proportion showed a directionally similar but nonsignificant omnibus effect (*F*_2,161_=2.57; *P*=.08; Figure 4), with poorer performance skewing toward more negative emotions and excellent performance toward more positive and neutral emotions. Cross-modal correspondence is evident: groups with higher negative emotions in SER also tended to have lower positive emotions in TER.

Across both modalities, the poor and excellent performance groups differed significantly for nearly all emotions.

**Figure 3.**
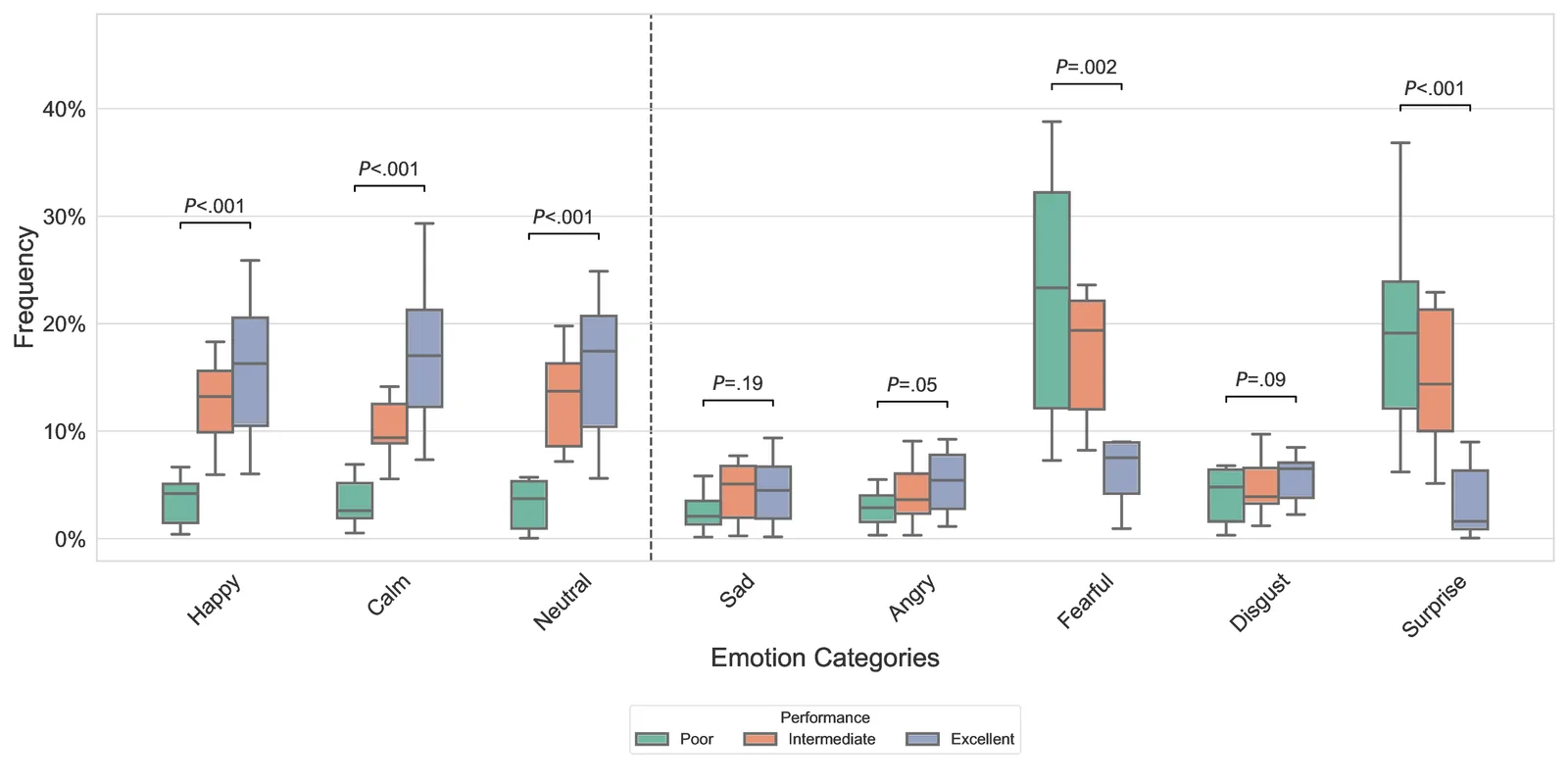
Speech-based emotion recognition (SER): emotion classification of participants’ speech and transcription according to their performance score group. The comparison evaluation is conducted between the poor and excellent groups.

**Figure 4.**
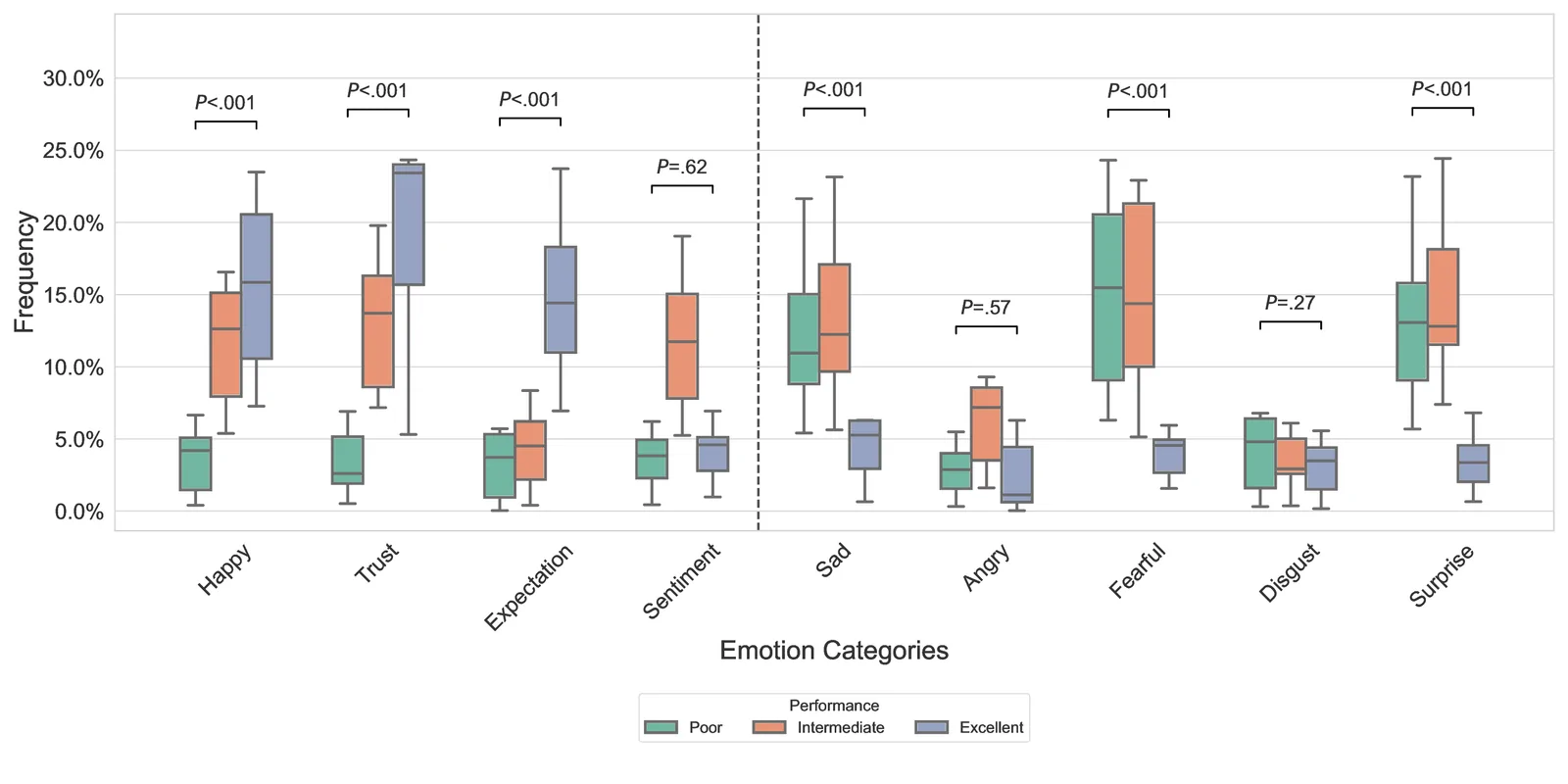
Text-based emotion recognition (TER): emotion classification of participants’ speech and transcription according to their performance group. The comparison evaluation is conducted between the poor and excellent groups.

### Sensitivity Analysis Using Mixed-Effects Models

Mixed-effects model results for speech- and text-based emotion proportions across performance groups are presented in Table 3. For SER, performance group remained significantly associated with happy, angry, fearful, surprise, calm, and neutral speech-emotion proportions after FDR correction. Sad and disgust were not significantly associated with performance group after correction. The largest excellent vs poor differences were observed for neutral speech affect, which was 4.24 percentage points higher in excellent performances; happy speech affect, which was 4.11 percentage points higher in excellent performances; and surprise speech affect, which was 3.17 percentage points lower in excellent performances.

**Table 3.** Mixed-effects sensitivity analysis of speech- and text-based emotion proportions across performance groups. Models were estimated separately for each emotion category, and emotion proportions were logit-transformed and modeled as the outcome, with performance group as the primary fixed effect. All models included random intercepts for resident and scenario and were adjusted for residency year and sex. Global performance group effects were evaluated using likelihood ratio tests comparing models with and without performance group. False discovery rate correction was applied across emotion-level tests within each modality.

Modality and emotion	LR^a^ χ²(2)	*P* value	FDR^b^–adjusted *P*	Excellent-poor difference
SER^c^				
Happy	22.03	<.001	<.001	4.11
Sad	2.19	.34	.38	−0.83
Angry	11.32	.003	.005	−1.66
Fearful	19.76	<.001	<.001	−2.31
Surprise	21.88	<.001	<.001	−3.17
Disgust	0.67	.72	.72	0.00
Calm	15.53	<.001	<.001	2.21
Neutral	25.71	<.001	<.001	4.24
TER^d^				
Happy	23.96	<.001	<.001	8.66
Sad	14.39	<.001	.003	−5.33
Angry	1.82	.40	.52	−0.89
Fearful	6.52	.04	.09	−2.91
Surprise	7.71	.02	.06	−4.13
Disgust	1.15	.56	.63	−0.15
Trust	3.67	.16	.24	2.06
Expectation	0.88	.64	.64	1.15
Sentiment	4.27	.12	.21	1.58

a LR: likelihood ratio.

b FDR: false discovery rate.

c SER: speech-based emotion recognition.

d TER: text-based emotion recognition.

For text-based emotion recognition, performance group remained significantly associated with happy and sad text-emotion proportions after FDR correction. Fearful and surprise text-emotion proportions showed nominal associations with performance group but did not remain significant after correction. Angry, disgust, trust, expectation, and sentiment were not significantly associated with performance group. The largest excellent vs poor differences were observed for happy text affect, which was 8.66 percentage points higher in excellent performances; sad text affect, which was 5.33 percentage points lower in excellent performances; and surprise text affect, which was 4.13 percentage points lower in excellent performances.

Together, these sensitivity analyses support the overall direction of the primary findings: higher-rated simulations showed higher positive, calm, or neutral affective patterns, whereas lower-rated simulations showed higher fearful or surprise-related affective patterns. However, the text-based findings were weaker and more emotion-specific than the speech-based findings, supporting the interpretation that speech- and text-based emotion recognition provide complementary but differently sensitive measures of affective communication.

### Affective Communication Across Simulation Phases

For this experiment, we compared the alignment (ie, agreement) between TER and SER across the simulation phase. For each phase, we allocated the 2 most prevalent emotion categories; if the highest-scoring emotion exceeded 50%, it was considered “dominant.” Phase-specific analyses revealed cross-modal concordance: negative SER and TER categories peaked during extreme deterioration and ACLS management, whereas calm and positive emotions predominated during the initial condition and recovery phases. Table 4 summarizes the top 2 categories per modality and phase; differences are most pronounced between the excellent and poor performance groups during high stress phases.

**Table 4.** Participants’ text-based emotion recognition (TER) and speech-based emotion recognition (SER) results with respect to the simulation phases. The percentage indicates the prevalence of emotions.

	Initial condition		Extreme deterioration		ACLS^a^ management		Recovery	
	Top 2 vocal emotions (%)	Top 2 textual emotions (%)	Top 2 vocal emotions (%)	Top 2 textual emotions (%)	Top 2 vocal emotions (%)	Top 2 textual emotions (%)	Top 2 vocal emotions (%)	Top 2 textual emotions (%)
Poor performance	*Fearful (50.03)^b^* Angry (31.78)	Fearful (36.70) Sad (30.22)	*Surprise (64.12)* Fearful (18.95)	*Surprise (59.81)* Fearful (14.08)	Angry (11.89) *Fearful (64.57)*	*Surprise (55.78)* Fearful (23.08)	Sad (30.73) Angry (24.67)	*Sad (50.89)* Surprise (26.84)
Intermediate performance	Calm (40.32) Neutral (16.70)	*Happy (52.13)* Trust (33.41)	*Calm (53.25*) Sad (23.97)	*Sad (50.22*) Sentiment (20.69)	Calm (44.19) Neutral (35.67)	Surprise (34.89) Expectation (32.22)	Happy (37.21) Calm (34.18)	*Happy (53.29)* Trust (30.62)
Excellent performance	*Calm (57.10*) Happy (27.85)	*Expectation (66.78*) Sentiment (11.67)	*Surprise (53.06*) Calm (22.65)	*Surprise (63.88)* Expectation (9.23)	Neutral (49.10) Calm (37.66)	*Expectation (50.77)* Happy (31.05)	*Neutral (53.28)* Happy (26.85)	*Happy (64.92)* Fearful (14.78)

a ACLS: advanced cardiovascular life support.

b Cases in which one of the emotions has a prevalence of over 50% (ie, “dominant”) are indicated in italics.

Across phases, 3 broad patterns emerged. First, differences between performance groups were most pronounced during the clinically demanding phases of extreme deterioration and ACLS management. Second, poor performance simulations showed a greater concentration of high-arousal negative emotion labels, particularly fear and surprise, during these phases. Third, excellent performance simulations more often showed calm, neutral, or positive affective labels during phases requiring coordinated action and recovery. Thus, the phase-based results suggest that affective communication differences were not uniform across the simulation but were most evident during moments of acute clinical demand.

## Discussion

### Principal Findings

In this prospective, simulation-based study of senior anesthesia residents, we observed systematic differences in model-derived affective communication patterns across performance groups. In the primary omnibus analysis, SER profiles differed across performance groups (*F*_2,161_=4.61; *P*=.02): residents in the poor performance group (scores ≤2) expressed predominantly fear and surprise, whereas those in the excellent performance group (score=5) showed higher proportions of calm, happy, and neutral speech affect. TER profiles showed a directionally similar but nonsignificant omnibus effect (*F*_2,161_=2.57; *P*=.08). Exploratory mixed-effects sensitivity analyses supported the overall direction of the speech-based findings. Performance group remained associated with happy, angry, fearful, surprise, calm, and neutral speech-emotion proportions after FDR correction. The largest excellent vs poor differences were observed for neutral speech affect (+4.24 percentage points), happy speech affect (+4.11 percentage points), and surprise speech affect (−3.17 percentage points). For text-based emotion recognition, performance group remained associated with happy and sad text-emotion proportions after correction, whereas fearful and surprise text-emotion proportions showed nominal associations that did not remain significant after correction. These findings suggest that speech-derived affective markers were more consistently associated with expert-rated performance than text-derived markers, although both modalities showed patterns consistent with the primary results.

### Interpretation of Affective Communication Patterns

The present study does not establish that emotion recognition outputs improve the accuracy, reliability, or predictive validity of simulation-based competency assessment. The findings show associations between model-derived affective communication patterns and expert-rated simulation performance. Therefore, these markers should be interpreted as exploratory and complementary signals that may inform formative feedback and future research, not as standalone assessment tools or substitutes for expert evaluation.

Phase-stratified summaries indicated that differences were most pronounced during extreme deterioration and ACLS management, when residents were required to recognize deterioration, coordinate the team, and execute time-sensitive actions. In these phases, higher-rated residents maintained calmer/neutral speech affect and more positive/neutral text tone. These associations suggest that affective communication patterns—how emotion is expressed and regulated—vary across performance groups and across the simulation phase. We interpret these findings as associations rather than causal effects. Elevated expression of emotions such as fear and surprise may co-occur with uncertainty or cognitive overload, whereas calm and neutral affect may co-occur with clearer commands, closed-loop confirmation, and anticipatory planning.

### Modality-Specific Findings: Speech vs Text

The discrepancy between SER and TER findings warrants careful interpretation. In the primary omnibus analysis, SER showed a statistically significant association with performance group, whereas TER showed a directionally similar but nonsignificant trend. This suggests that speech-derived affective information may have been more robustly captured than text-derived affective information in the present dataset. One explanation is that SER captures paralinguistic features, including pitch, intensity, tempo, pauses, hesitation, and vocal arousal, which may be especially informative during high-stress simulation. TER, in contrast, depends on a multistep pipeline; thus, errors or biases introduced at any stage may attenuate the estimates.

Several modality-specific limitations may have contributed to the weaker primary TER finding. Residents’ speech during crisis management was often brief, imperative, protocol-driven, and interspersed with Hebrew-English clinical terminology. Such utterances may contain limited lexical emotional information, even when vocal delivery conveys strong affective cues. Automatic transcription errors, word-level timestamping errors, code-switching, medical abbreviations, and domain-specific terminology may also introduce noise. In addition, TER models may misclassify clinically appropriate urgency, direct commands, or technical language as negative affect, whereas SER models may misinterpret raised volume or rapid speech as distress even when these features reflect appropriate leadership. Therefore, neither modality should be treated as a direct measure of psychological state or competency.

The mixed-effects sensitivity analyses suggested that some TER categories, particularly happy and sad, varied across performance groups after adjustment for repeated simulations and scenario-level clustering. However, these analyses were exploratory and emotion-specific; therefore, they should be interpreted as hypothesis-generating. Overall, the findings suggest that SER and TER provide complementary but imperfect views of affective communication, with different sources of noise, bias, and context dependence.

### Educational Implications

Clinically, the phase specificity matters. The clearest separation between excellent and poor performances emerged precisely when the team, specifically the resident, transitioned from recognizing deterioration to taking decisive action. That observation generates practical hypotheses for training: debriefings might explicitly review affective communication during these windows; phase-targeted coaching could rehearse calm and neutral delivery of closed-loop commands; and formative feedback could pair technical checklists with brief, objective summaries of affective patterns.

From an educational perspective, the most appropriate near-term use of these metrics is formative rather than summative. SER and TER outputs should not be used to make independent pass-fail decisions, rank residents, or replace expert faculty assessment. Instead, they may be used as debriefing aids that help educators identify specific moments in a simulation where affective communication changed. For example, an educator could review a timeline showing increases in high-arousal vocal affect, pauses, or shifts toward calmer communication and use these moments to guide reflective discussion about the clinical context, communication clarity, use of closed-loop communication, and whether the emotional tone supported or interfered with team coordination.

### Limitations and Future Directions

Several important limitations should be considered when interpreting these findings. First, this was an exploratory observational study; therefore, the results cannot determine whether the observed affective patterns reflect competency-relevant communication, cognitive load, stress response, individual communication style, or other latent constructs. Future studies should combine automated emotion recognition with expert-coded communication behaviors, physiological stress measures, and multi-rater performance assessments to clarify these mechanisms. Second, the primary analyses were univariate and did not fully adjust for potential confounders, including sex, postgraduate year, scenario, site, and language-related factors. Although we added exploratory mixed-effects sensitivity analyses, these models were not prespecified and should be interpreted as robustness checks rather than definitive confirmatory analyses. Future studies should use preregistered analytic plans, fully powered hierarchical models, and richer covariate structures to account for repeated simulations, scenario-level clustering, hospital affiliation, and other potential confounders.

Third, performance ratings were primarily based on single-rater expert assessments. Therefore, these ratings should be viewed as expert-rated indicators of simulation performance rather than definitive reference standards for clinical competency. Future work should incorporate multiple independent raters, standardized rating procedures, rater training, and reliability analyses to strengthen the validity of the performance outcome.

Fourth, SER may be affected by background alarms, overlapping speech, microphone placement, speech separation errors, speaker-specific vocal characteristics, and cultural or language-specific differences in vocal affect. TER may be affected by transcription errors, segmentation errors, code-switching, short utterance length, clinical abbreviations, and limited lexical emotional content in protocol-driven commands. These sources of noise may differentially affect SER and TER, partly explaining why the primary SER findings were stronger than the primary TER findings.

Additionally, the models operated on Hebrew speech with Hebrew-English code-switching, which may have amplified speech-to-text and emotion classification errors. While we accounted for domain adaptation in medical discourse, a known challenge given specialized vocabulary and context-dependent meaning [27], we did not specifically adapt the models to the emotional and communicative context of anesthesiology crisis simulation.

Finally, time-aligned analyses could further examine whether short surges in high arousal negative affect precede delayed or missed actions, and whether calm or neutral affect during extreme deterioration and ACLS management is associated with more timely execution after adjustment for resident-, site-, and scenario-level factors.

### Conclusions

In conclusion, this exploratory observational study suggests that automated speech- and text-based emotion recognition can capture phase-specific affective communication patterns during anesthesiology critical incident simulation training. Speech-derived emotion markers, and to a lesser extent, text-derived markers, were associated with expert-rated performance groups. These findings should not be interpreted as evidence that emotion recognition can independently assess competency or improve assessment accuracy. Rather, they support the feasibility of using affective communication markers as complementary tools for simulation debriefing, formative feedback, and future research on communication under crisis conditions. Further validation using multirater outcomes, adjusted statistical models, and larger multicenter datasets is needed before such measures can inform competency assessment.

## Abbreviations

ACLS: advanced cardiovascular life support
ATLS: advanced trauma life support
EEND-SS: end-to-end neural diarization with speaker subspace
FDR: false discovery rate
GPU: graphics processing unit
ICU: intensive care unit
NLP: natural language processing
OR: operating room
SBA: simulation-based assessment
SER: speech-based emotion recognition
TER: text-based emotion recognition
